# Brandy as a Medicine

**Published:** 1870-01

**Authors:** 


### BRANDY AS A MEDICINE.

Brandy kills multitudes every year who enjoyed perfect health before they began to use it; hence it would seem fair to infer that it will kill the sick much more speedily.

Dr. Lees said that he was living near Buckingham Palace, in London, when Prince Albert was taken sick. His case was doing well for a few days, when they began to give him brandy to strengthen him, to enable him to recover more rapidly; the more he was stimulated the worse he grew until he died. It is true that they believed it was the best thing for him, but their thinking so did not make it so.

Some years ago, when it was the custom to attempt curing

DELIRIUM TREMENS

by giving brandy, one out of every four thus treated died at the Edinburgh Hospital. Since then the professor in the medical department there has treated three hundred cases of delirium tremens, without alcohol, without losing a single patient.

Professor Gardner, of the Glasgow University, gave a hundred men thirty ounces of alcohol; seventeen died out of the hundred. Another hundred were allowed three ounces, and eleven died out out of the hundred.

Of two hundred and nine cases of young persons, who were not allowed either wine or whisky, not one died.

In a teetotal hospital at Leeds, of three hundred patients who took not a drop, all recovered. Let facts decide.

